# Myofibroblasts impair myocardial impulse propagation by heterocellular connexin43 gap-junctional coupling through micropores

**DOI:** 10.3389/fphys.2024.1352911

**Published:** 2024-02-23

**Authors:** Yumika Tsuji, Takehiro Ogata, Kentaro Mochizuki, Shoko Tamura, Yuma Morishita, Tetsuro Takamatsu, Satoaki Matoba, Hideo Tanaka

**Affiliations:** ^1^ Department of Pathology and Cell Regulation and, Graduate School of Medical Science, Kyoto Prefectural University of Medicine, Kyoto, Japan; ^2^ Department of Cardiovascular Medicine, Graduate School of Medical Science, Kyoto Prefectural University of Medicine, Kyoto, Japan; ^3^ Department of Medical Photonics, Kyoto Prefectural University of Medicine, Kyoto, Japan; ^4^ Faculty of Health and Medical Sciences, Kyoto University of Advanced Science, Kyoto, Japan

**Keywords:** myofibroblast, cardiomyocyte, optical mapping, impulse propagation, electrotonic effect, connexin 43, cardiac arrhythmia, microporous membrane

## Abstract

**Aim:** Composite population of myofibroblasts (MFs) within myocardial tissue is known to alter impulse propagation, leading to arrhythmias. However, it remains unclear whether and how MFs alter their propagation patterns when contacting cardiomyocytes (CMs) without complex structural insertions in the myocardium. We attempted to unveil the effects of the one-sided, heterocellular CM-MF connection on the impulse propagation of CM monolayers without the spatial insertion of MFs as an electrical or mechanical obstacle.

**Methods and results:** We evaluated fluo8-based spatiotemporal patterns in impulse propagation of neonatal rat CM monolayers cultured on the microporous membrane having 8-μm diameter pores with co-culture of MFs or CMs on the reverse membrane side (CM-MF model or CM-CM model, respectively). During consecutive pacing at 1 or 2 Hz, the CM monolayers exhibited forward impulse propagation from the pacing site with a slower conduction velocity (*θ*) and a larger coefficient of directional *θ* variation in the CM-MF model than that in the CM-CM model in a frequency-dependent manner (2 Hz >1 Hz). The localized placement of an MF cluster on the reverse side resulted in an abrupt segmental depression of the impulse propagation of the upper CM layer, causing a spatiotemporally non-uniform pattern. Dye transfer of the calcein loaded in the upper CM layer to the lower MF layer was attenuated by the gap-junction inhibitor heptanol. Immunocytochemistry identified definitive connexin 43 (Cx43) between the CMs and MFs in the membrane pores. MF-selective Cx43 knockdown in the MF layer improved both the velocity and uniformity of propagation in the CM monolayer.

**Conclusion:** Heterocellular Cx43 gap junction coupling of CMs with MFs alters the spatiotemporal patterns of myocardial impulse propagation, even in the absence of spatially interjacent and mechanosensitive modulations by MFs. Moreover, MFs can promote pro-arrhythmogenic impulse propagation when in face-to-face contact with the myocardium that arises in the healing infarct border zone.

## 1 Introduction

Cardiac myofibroblasts (MFs), which proliferate in response to various pathological insults to the heart (Cartledge et al., 2021), have attracted attention as important players in the development of arrhythmias (Kohl and Camelliti, 2012; Rohr, 2012). Several *in vivo* studies have suggested that MFs contribute to arrhythmogenic slowing of impulse propagation in the granulation tissue of infarcted heart (Walker et al., 2007; Mahoney et al., 2016; Rubart et al., 2018). However, the detailed mechanisms underlying arrhythmogenesis of MFs in heart tissue have been poorly elucidated because of their structural complexities and the difficulty in precisely characterizing cellular electrophysiological changes. In contrast, from *in vitro* cell culture studies, growing evidence has accumulated on the mechanisms underlying the arrhythmogenesis of MFs (Kohl and Camelliti, 2012; Rohr, 2012) owing to simple and highly reproducible systems (Bub et al., 2003; Tung and Zhang, 2006). For example, by using neonatal rat cardiomyocyte (CM) monolayers mixed with MFs (Zlochiver et al., 2008; Askar et al., 2011) or synthetic CM strands overlaid by MFs (Gaudesius et al., 2003; Miragoli et al., 2006; Miragoli et al., 2007), the slowing of impulse propagation (Gaudesius et al., 2003; Miragoli et al., 2006; Zlochiver et al., 2008; Askar et al., 2011; Askar et al., 2012), development of ectopic impulse (Miragoli et al., 2007), and generation of re-entrant tachyarrhythmias (Zlochiver et al., 2008; Askar et al., 2011; Askar et al., 2012) have been established. Additionally, most arrhythmogenic impulse alterations depend on the amount of MF (Miragoli et al., 2006; Zlochiver et al., 2008) and are mediated by heterocellular connexin 43 (Cx43) gap-junctional coupling between CMs and MFs (Miragoli et al., 2006; Zlochiver et al., 2008; Askar et al., 2011). These experimental studies are entirely based on the electrophysiological interactions of CMs and MFs; CMs are depolarized electrotonically by coupling with the less polarized MFs via gap junctions (Rook et al., 1992), resulting in the depression of the impulse conduction by reduction in the Na^+^ channel availability (Miragoli et al., 2006). However, these heterocellular culture systems still involve complex patterns of interaction between CMs and MFs (Kohl and Camelliti, 2007): i) zero-sided connection showing no gap-junctional coupling of MFs with CMs acting as electrical insulators, ii) single-sided connection showing electrotonic coupling of CMs by direct attachment to MFs, and iii) double-sided connection of CMs with interposed MFs causing an insert-length-dependent impulse propagation delay (Gaudesius et al., 2003). The CM monolayer models, which incorporate mixed co-cultured with MFs (Zlochiver et al., 2008; Askar et al., 2011; Askar et al., 2012) include all these patterns of heterocellular connections, where MFs infiltrate randomly among CMs, acting as a passive electrical barrier of conduction, and thereby become proarrhythmogenic by zigzag impulse propagation, as observed in infarcted hearts (De Bakker et al., 1993). Moreover, mechanical stretch (Kamkin et al., 2005; Thompson et al., 2011) and α-smooth muscle actin stress fibers (Rosker et al., 2011) of MFs that activate mechanosensitive channels of CMs (Kamkin et al., 2005), which have been suggested to contribute to arrhythmogenesis, may also be involved in these composite models. The placement models of MFs over the thin strand of CMs (Miragoli et al., 2006; Miragoli et al., 2007) or on the 2D monolayer of CMs (Rosker et al., 2011) do not incorporate zero-sided, or double-sided connection; however, these models include both the single-sided connection and mechanical connection between these two cell types, leading to a conduction delay (Gaudesius et al., 2003; Grand et al., 2014). In addition to the cultured-cell studies, computer simulation studies have also made great contributions to understanding the electrophysiological basis for CM-MF connections (Jacquemet and Henriquez, 2008; Xie et al., 2009); however, the role of mechanosensitive aspects in the electrical interactions of MFs with CMs has been scarecely considered.

Therefore, it needs to be clarified whether and how the pattern of impulse propagation is altered when CMs connect with MFs without a random mixture and mechanosensitive interactions of these two cell types in the myocardium. To address this issue, we performed monolayer cultures of neonatal rat CMs on membranes rich in 8-µm diameter micropores with MFs co-cultured on the reverse membrane side and conducted fluo8-based spatiotemporal imaging of impulse propagation in the CM monolayers. Our results provide definitive evidence for a single-sided interface of MFs and CMs in the genesis of proarrhythmic impulse propagation even in the absence of mechano-sensitive interactions between these two types of cells.

## 2 Materials and methods

### 2.1 Regents and materials

All reagents and materials used in this study are listed in Table 1.

**TABLE 1:** The reagents and materials.

Product name	Catalog number	Manufacturers
Cell culture inserts	353,093, 353,182	Corning
Collagenase Type II	CLS2	Worthington Biochemical Corp.
Fluo8-AM	ab142773	Abcam
rabbit monoclonal antibody against alpha smooth muscle actin (αSMA)	ab150301	Abcam
mouse monoclonal antibody against GAPDH	ab8245, ab105428	Abcam
rabbit polyclonal antibody against Cx43 (Gja1)	C6219	Sigma-Aldrich
mouse monoclonal antibodies against vimentin	V6630	Sigma-Aldrich
mouse monoclonal antibodies against troponin T (Clone 13-11)	MA5-12960	Thermo Fisher Scientific Inc.
Alexa Fluor 488 goat anti-rabbit IgG and goat anti-mouse IgG	A11008, A11001	Thermo Fisher Scientific Inc.
Alexa Fluor 633 goat anti-mouse IgG	A21052	
Alexa Fluor 555 goat anti-rat IgG and goat anti-mouse IgG	A21434, A21422	
rat monoclonal antibody against vimentin	NL2105R	R&D SYSTEMS
DAPI Fluoromount-G^®^	0100-20	Southern Biotech
Calcein-AM solution	C396	DOJINDO
heptanol	17,610-32	Nacalai Tesque, Inc.
Stealth RNAi™ siRNA against rat *Gja1*	RSS351267	Thermo Fisher Scientific Inc.
Stealth RNAi™ siRNA Negative Control	12,935,300	Thermo Fisher Scientific Inc.
Lipofectamine^®^ RNAiMAX transfection reagent	13,778,075	Thermo Fisher Scientific Inc.

### 2.2 Animals

Wistar rats were purchased from Japan SLC. Inc. (Shizuoka, Japan). All the animal experiments described in this study were performed in accordance with the ARRIVE guidelines 2.0 (https://arriveguidelines.org) and the Guide for the Care and Use of Laboratory Animals (8th edition, National Academies Press, Washington DC, 2011) following approval by the Institutional Animal Care and Use Committee of Kyoto Prefectural University of Medicine (Approval No. M2022-207).

### 2.3 Cell culture

**FIGURE 1:**
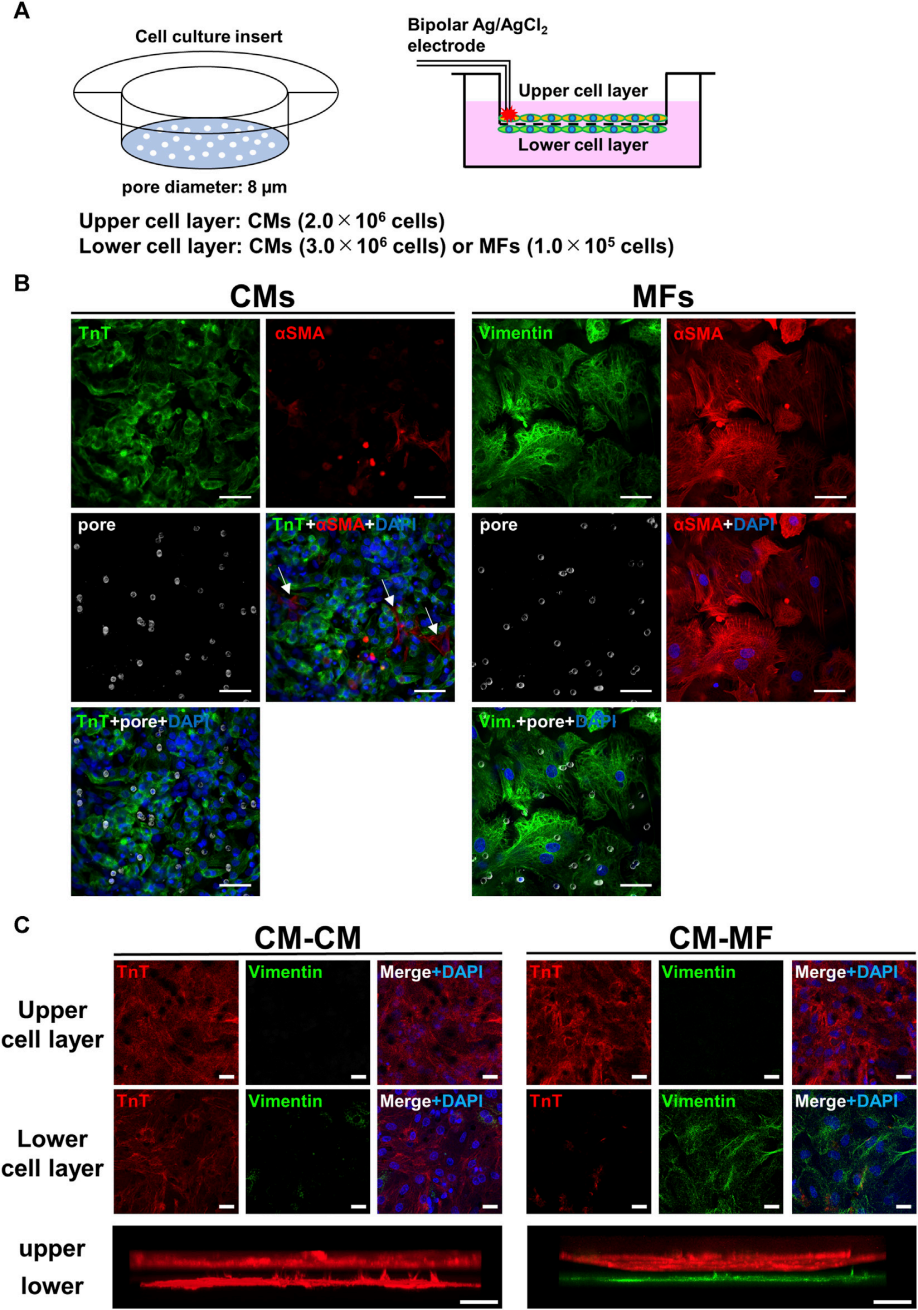
Overview of two-layer cell culture system using cell culture inserts. **(A)** The schematic illustrations for the cell culture inserts having 8-μm diameter pores (left panel) and the cell-culture model formed on the upper and lower layers separated by a microporous membrane (right panel). Bipolar silver chloride electrodes placed at the rim of the upper cell layer are for electrical stimulation. **(B)** Confocal immunofluorescence images of cardiomyocytes (CMs) and myofibroblasts (MFs) monolayers cultured on the cell culture insert and distributions of micropores on the membrane visualized by inverted DIC images obtained simultaneously. Blue signals denote nuclei stained with DAPI and white circles membrane pores. Troponin T (TnT) was immunostained for CMs, and vimentin and αSMA were for MFs. The scale bar = 50 μm. **(C)** Confocal immunofluorescence images of the upper and lower cell layers for CM-CM (left panel) and CM-MF (right panel) models. Lower panels show the respective side-view, stack images. The scale bar = 20 μm.

Ventricular CMs and MFs were obtained from 1-day-old neonatal Wistar rat hearts for primary culture (Nakagami et al., 2008). Two nests of neonatal rats were euthanized by an overdose of isoflurane anesthesia (5% concentration), and subsequently the hearts were excised and exsanguinated for enzymatic isolation of ventricular cardiomyocytes. We every time conducted cell isolations from 2 nests at once, and in total 18 cases of isolation were performed from 36 nests. After mincing the isolated hearts, the CMs and ventricular fibroblasts were dissociated with 0.2% collagenase (Type II, CLS2), resuspended in DMEM containing 10% FBS, and differentially sorted using two pre-plating steps (20 min each). The isolated fibroblasts were cultured in advance for 1 week in a plastic Petri dish (92 mm diameter, Falcon^®^); consequently, we obtained activated fibroblasts MFs rather than fibroblasts. On the upper side of Falcon^®^ Cell Culture Inserts (Permeable Support for 6-well plate Transparent PET membrane with 8.0-µm diameter micropores, Figure 1A, left panel), CMs were plated. We seeded a high density (2.0 × 10^6^ cells) of CMs on the membrane to make a confluent monolayer. Under these conditions intercellular gap formation and fibroblast proliferation were prevented through contact inhibition. Before seeding the CMs on the upper membrane side, the culture insert was inverted. On the reverse side, either MFs (1.0 × 10^5^ cells) or CMs (3.0 × 10^6^ cells) were seeded, and subsequently incubated for 4 days in DMEM solution containing 10% FBS with no BrdU added, referred respectively as the CM-MF model or CM-CM model (Figure 1A, right panel) for the experiments.

### 2.4 Localized spot pattern cell culture on the lower layer

CMs (2.0 × 10^6^ cells) were cultured uniformly on the upper side of the cell culture inserts to create confluent monolayer of CMs. Spot cell seeding preparations were created by seeding of either MFs (1.0 × 10^4^ cells or 4.0 × 10^4^ cells) or CMs (1.0 × 10^6^ cells) in a localized spot pattern on the reverse side of the membrane using a 10-mm inner diameter silicone cylinder (Figure 3A). After 4 days of cell seeding the preparation was used for experiments.

### 2.5 Ca^2+^ imaging of the impulse propagation in the upper CM monolayer

The CMs in the upper layers were incubated for 15 min at 37°C with fluo8-AM (5 μM) in Tyrode solution consisting of 137 mM NaCl, 4 mM KCl, 1.2 mM CaCl_2_, 1.0 mM MgCl_2_, 0.33 mM NaH_2_PO_4_, 10 mM HEPES, and 10 mM glucose (pH 7.4 adjusted with NaOH). After washout of the extracellular fluo8-AM, the cell culture inserts were placed on a perfusion chamber filled with Tyrode solution and maintained at 33°C by a temperature control device under constant superfusion at 3.3 mL/min. Cell culture inserts were uniformly exposed to blue LED light (465 ± 20 nm) from three pieces of emitter (CCS, Kyoto, Japan) to excite fluo8-loaded samples, and the emitted fluorescence was detected through a filter cube (U-MGFPHQ, Olympus), which incorporates a dichroic mirror with a cut-off wavelength of 485 nm and an emission filter with a transparent wavelength region of 495–540 nm.

We detected spatiotemporal changes in the fluo8-fluorescence intensity of CMs to evaluate the pattern of impulse propagation of the upper CM monolayer using a macro zoom fluorescence microscope (MVX10 MacroView; Olympus, Tokyo, Japan) (Supplementary Figure S1) equipped with charge-coupled device (CCD) camera (MiCaM02-HR; Brainvision Inc., Japan) as previously demonstrated (Nakagami et al., 2008). We analyzed the changes in the fluo8-fluorescence intensity because the dynamic range of the Ca^2+^ transients (Supplementary Figure S1) is much greater than the voltage-sensitive dye-based action potentials. We analyzed the spatiotemporal images of the fluo8-fluorescence intensity of the CMs on the upper membrane layer using two modes of acquisition. For X-t data the images were acquired from a field-of-view of 30 × 22 mm (40 × 28 pixels) at a sampling rate of 333 frames/s. For isochronal maps images were from a field-of-view of 30 × 22 mm (184 × 124 pixels) at a sampling rate of 62.5 frames/s. These two modes of image acquisitions were conducted at the same field of view, i.e., the same size. Ca^2+^ transients of CMs on the upper layer were evoked by electrical point stimulation with biphasic pulses of 1-ms duration and 1.5 times the threshold current of excitation at the rim of the culture insert with bipolar silver chloride electrodes (0.5 mm diameter) applied at 1 and 2 Hz (Figure 1A, right panel).

### 2.6 Data analysis of Fluo8-fluorescence images

We evaluated the spatiotemporal changes in fluo8-fluorescence intensity of the impulse propagation of the upper CM layer using a MiCAM02 data analysis system (Brainvision, Inc., Tokyo, Japan). For quantitative analysis, we adopted preparations of CMs that showed Ca^2+^ transients entrained by consecutive pacing at 1 Hz and 2 Hz. The conduction velocity (*θ*) was calculated from the slope of the X-t images; that is, the changes in the fluo8-fluorescence intensity along a free straight line drawn in the wavefront direction were divided by the elapsed time (middle panel in Supplementary Figure S1). The spatiotemporal uniformity of the impulse propagation was evaluated using the coefficient of directional variation of *θ* (CV_*θ*_) obtained at a 15-degree interval (bottom panel in Supplementary Figure S1). The CV_*θ*_ was obtained by dividing the respective *θ* values by the standard deviation (SD) of the mean *θ*. An isochronal map was created from the stored fluorescence images.

### 2.7 Immunostaining

Cells cultured on the culture inserts were fixed in ice-cold acetone, and the insert membrane was directly immunostained. Anti-Gja1/Cx43 antibody (1:2000) was used as the primary antibody, and Alexa Fluor 488 goat anti-rabbit IgG (1:500) was used as the secondary antibody. Cells were identified with anti-cardiac troponin T (1:1000) for CMs and anti-vimentin (1:200) or α-smooth muscle actin (αSMA) (1:500) for MFs as the primary antibodies and Alexa Fluor 633, 488, or 555 goat anti-mouse IgG, Alexa Fluor 555 goat anti-rat IgG antibodies, and Alexa Fluor 488 goat anti-rabbit IgG as the secondary antibodies. Fluorescence images of the immunostained cells and the differential interference contrast (DIC) images of micropores were acquired by a confocal fluorescence microscope (LSM900, ZEISS). The mesoscopic fluorescence images (Figure 3A) were obtained by a wide-field-based fluorescence microscope (BZ-X800, KEYENCE). The cell nuclei were stained with DAPI Fluoromount-G^®^. To measure the area size of cells, we cultured relatively low numbers of CMs and MFs (1 × 10^4^ cells) on the 35 mm-diameter plastic dish. The contour of each cell body was manually outlined, and the internal area size was then calculated using the Fiji software (Schindelin et al., 2012).

### 2.8 Calcein dye loading

CMs were plated on the upper side of Falcon^®^ Cell Culture Inserts (Permeable Support for 12-well Plate Transparent PET Membrane with 8.0-µm diameter micropores). CMs (7.0 × 10^5^ cells) were then cultured on the upper side of the cell culture insert, and either MFs (3.0 × 10^4^ cells) or CMs (1.0 × 10^6^ cells) were cultured on the reverse membrane side in DMEM containing 10% FBS for 4 days. For some samples, the HeLa cells were cultured in the reverse side. Calcein-AM dye (1 μg/mL) was incorporated into the CMs on the upper membrane side after 4-day culture on the cell culture inserts and incubated for about 5 s. After washing the extracellular calcein-AM, images of the fluorescence intensity of intracellular calcein (excitation wavelength, 490 nm; emission wavelength, 525 nm) were obtained on both sides of the culture insert membrane using a confocal microscope (LSM900, ZEISS). Heptanol (0.5 mM) was used to inhibit gap junctional communication.

### 2.9 Gene silencing by siRNA interference

*Gja1* was silenced using RNA interference. According to the manufacturer’s protocol, Stealth RNAi™ siRNA targeting *Gja1* (siRNA) or negative control was transfected into MFs the day after the start of culture. The transfection medium was removed after 48 h of treatment.

### 2.10 Western blotting

Cell lysates were extracted using a lysis buffer (50 mM Tris-HCl, pH 7.5, 150 mM NaCl, 50 mM EDTA, 1% Triton X-100, and protease-phosphatase inhibitor mixture). Protein samples were subjected to sodium dodecyl sulfate-polyacrylamide gel electrophoresis and transferred to polyvinylidene difluoride membranes, which were then incubated with the primary antibodies against Cx43 or GAPDH. Horseradish peroxidase-conjugated anti-rabbit or anti-mouse IgG (GE Healthcare) was used as the secondary antibody.

### 2.11 Statistical analysis

All experiments were performed at least three times unless otherwise stated. Values are expressed as means ± SD in the text. In the graph medians ± quartiles (25% and 75%) with means are depicted in box plots with Tukey whiskers unless otherwise stated. Statistical analyses were performed using the GraphPad Prism 8 statistical package (GraphPad Software Inc.). In statistical analyses, we first performed the Kolmogorov–Smirnov assumption test for normal data distribution. If the data were normally distributed, we conducted Wilcoxon signed rank test for paired data, and Student’s t-test for unpaired data. For unpaired non-normally distributed data we used the Mann–Whitney *U* test. The Fisher’s exact test was used in the analysis of contingency tables. Differences were considered statistically significant at *p* < 0.05.

## 3 Results

### 3.1 MFs on the reverse membrane side slow the conduction velocity and impair the uniformity of the impulse propagation of the CM monolayer

Figure 1B shows the immunofluorescence images for CMs and MFs on the upper layer, and distributions of micropores on the membrane visualized by inverted DIC images obtained simultaneously. The merged images of the membrane pores and nuclei by DAPI with either the troponin T (TnT)-positive cell layer for CMs or vimentin- or α-smooth muscle actin (αSMA)-positive cell layer for MFs revealed that most of the pores were covered by cells. The averaged surface areas of individual cells were estimated to be 1.3 × 10^3^ μm^2^ (n = 10 cells) for CMs and 8.5 × 10^3^ μm^2^ for MFs (n = 10 cells). These estimated area of CMs was comparable to the value of ∼1.5 × 10^3^ μm^2^ reported previously (Jousset et al., 2016), whereas the area of MFs was larger than the reported value of ∼5.0 × 10^3^ μm^2^ (Jousset et al., 2016). The distribution of micropores on the membrane was not uniform on the cellular scale having a mean density and standard deviation of 5.8 × 10^2^ ± 2.3 × 10^2^ pore/mm^2^ on the area of 93 μm × 93 µm (n = 6 regions), whereas the value on the near-millimeter-scale converged uniformly (5.7 × 10^2^ ± 2.9 × 10 pore/mm^2^ on the area of 930 μm × 930 μm, n = 6 regions). Representative allocations of pores to each single cell were approximately 0.2 pores for CM and 2.2 pores for MF, which were calculated from the area size of 465 µm × 465 µm. We also conducted immunocytochemistry of the cell layers on the upper and lower sides of the membrane. The CMs and MFs were immunocytochemically confirmed to be accurately cultured in each layer by TnT and vimentin, respectively (Figure 1C). We also confirmed that HeLa cells were accurately cultured on the lower layer of the membrane (Supplementary Figure S2A).

**FIGURE 2:**
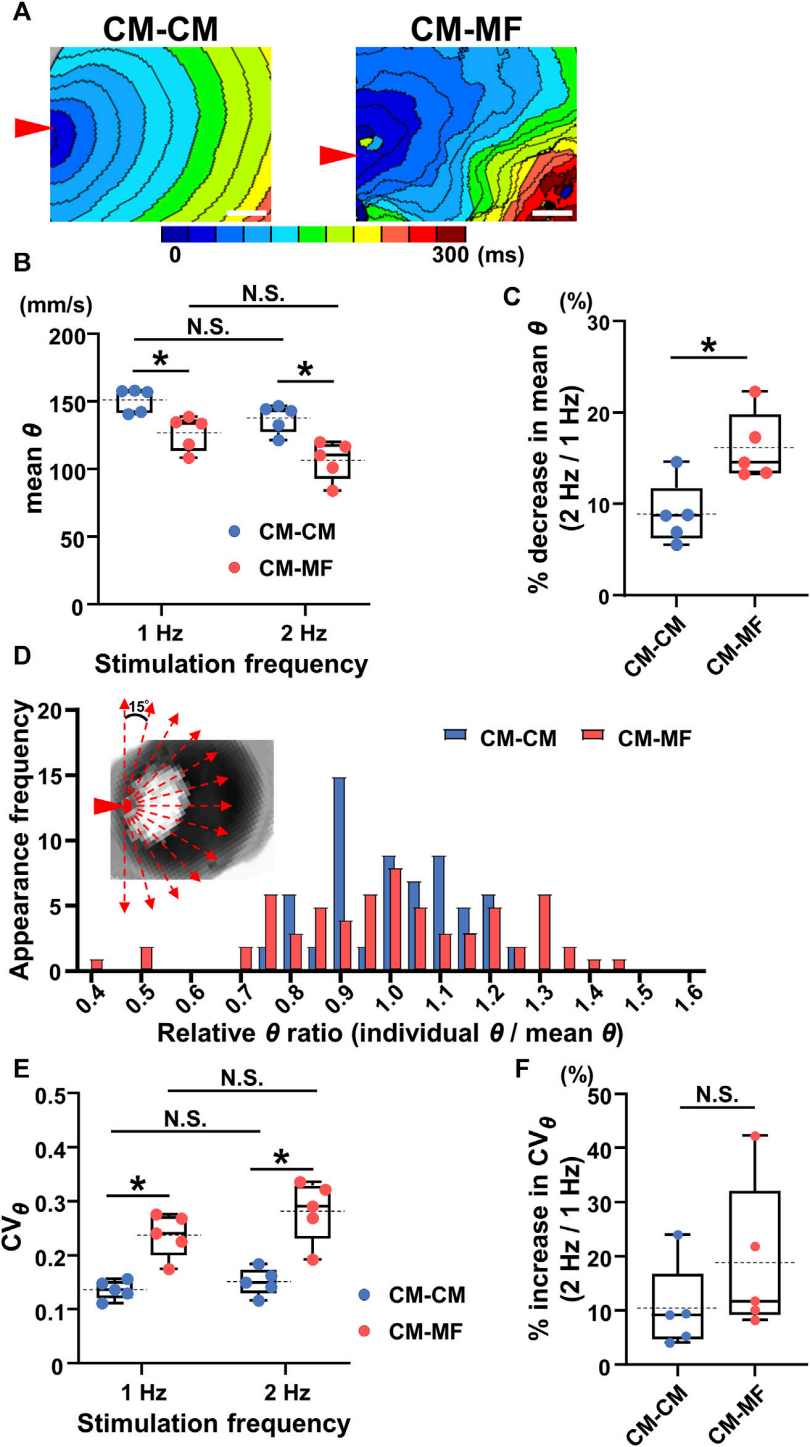
Myofibroblasts (MFs) on the reverse membrane side reduce the conduction velocity and impair its directional uniformity in the cardiomyocyte (CM) monolayer. **(A)** The representative isochronal maps in the CM-CM and CM-MF models. Red arrowheads denote the point of electrical stimulation. The white scale bar = 5 mm. **(B)** The mean conduction velocity (mean *θ*) of the upper CM layer in the CM-CM (blue dots) and CM-MF (red dots) models during 1- and 2-Hz pacing (CM-CM: n = 5/group, obtained from 4 isolations; CM-MF: n = 5/group, obtained from 5 isolations). **(C)** The percent decrease in mean values of *θ* as stimulation frequency increased from 1 Hz to 2 Hz in CM-CM and CM-MF models (n = 5 for each group corresponding to **(B)**. **(D)** The histogram of the ratios for relative conduction velocity (*θ*) in the CM-CM and CM-MF models. Inset shows the directions for measurements of the conduction velocity (also shown in Supplementary Figure S1). **(E)** The coefficient of variation (CV_*θ*_) of the mean conduction velocity (mean *θ*) of the upper CM layer during 1- and 2-Hz pacing in the CM-CM (blue dots) and CM-MF (red dots) models obtained from the same preparations as B (n = 5 for each group). **(F)** The percent increase in the variability of *θ* (CV_*θ*_) as the pacing frequency increased from 1 Hz to 2 Hz (n = 5 for each group corresponding to **(E)**. Data in **(B,C,E,F)** are box-plotted shown as median ± quartiles (25% and 75%). Mean values are depicted on the graphs as horizontal dashed lines. *p < 0.05. N.S. = not significant.

The isochronal maps at 1-Hz pacing demonstrated that the impulse propagation of the CM layer was slower in the CM-MF model than that in the CM-CM model (Figure 2A). Moreover, while the CM-CM model showed spatiotemporally uniform propagation, the CM-MF one showed irregular pattern. Quantitatively, the mean conduction velocity (*θ*) was significantly slower in the CM-MF model than in the CM-CM model both at 1- and 2-Hz pacing (at 1 Hz, CM-CM: 151.3 ± 8.9 mm/s [n = 5] vs. CM-MF: 126.9 ± 12.8 mm/s [n = 5], *p* = 0.0079; at 2 Hz, CM-CM: 137.8 ± 10.6 mm/s [n = 5] vs. CM-MF: 106.6 ± 14.4 mm/s [n = 5], *p* = 0.0079) (Figure 2B). There was no significant decrease in *θ* values between the two stimulation frequencies at 1 Hz and 2 Hz (Figure 2B, *p* = 0.0625, each); however, the percent change in the mean *θ* showed a more remarkable decrease in the CM-MF model than in the CM-CM model when the stimulation frequency was increased from 1 Hz to 2 Hz (CM-CM: 8.95% ± 3.46% [n = 5] vs. CM-MF: 16.19% ± 3.78% [n = 5], *p* = 0.0134) (Figure 2C). The mean *θ* values of the upper CM layer were not different among the CM-CM model, CM-HeLa model co-cultured with communication-deficient HeLa cells on the lower layer, and CM-no cell model in which no cells were cultured on the lower layer (CM-CM: 151.3 ± 8.9 mm/s [n = 5], CM-HeLa: 152.3 ± 9.6 mm/s [n = 5], CM-no cell: 150.5 ± 7.9 mm/s [n = 5]) (Supplementary Figure S2B).

The histogram of the *θ* ratio relative to the mean *θ* showed a larger variation in the CM-MF model than that in the CM-CM model (Figure 2D). During 1- and 2-Hz pacing, the coefficient of directional variation for *θ* (CV_*θ*_) was larger in the CM-MF than in the CM-CM model (1 Hz: CM-CM 0.136 ± 0.018 [n = 5] vs. CM-MF 0.237 ± 0.040 [n = 5], *p* = 0.0079; 2 Hz: CM-CM 0.151 ± 0.025 [n = 5] vs. CM-MF 0.282 ± 0.057 [n = 5], *p* = 0.0079) (Figure 2E), but there was no frequency-dependent changes in CV_*θ*_ between 1 Hz and 2 Hz in both the CM-CM model and CM-MF model (Figure 2E 1 Hz: 0.237 ± 0.040 [n = 5] vs. 2 Hz: 0.282 ± 0.057 [n = 5], *p* = 0.0625 for each). The percent increase in the CV_*θ*_ on increasing the pacing frequency from 1 Hz to 2 Hz was not significantly different between the CM-MF model and the CM-CM model (CM-CM: 10.40% ± 7.97% [n = 5] vs. CM-MF: 18.84% ± 14.12% [n = 5], *p* = 0.278) (Figure 2F). There was also no significant difference in the CV_*θ*_ among the CM-CM, CM-HeLa, and CM-no cell models (at 1 Hz, CM-CM: 0.136 ± 0.018, CM-HeLa: 0.117 ± 0.021, CM-no cell: 0.119 ± 0.027; n = 5 each) (Supplementary Figure S2C).

### 3.2 Local placement of MFs causes local propagation slowing of the CM monolayer

**FIGURE 3:**
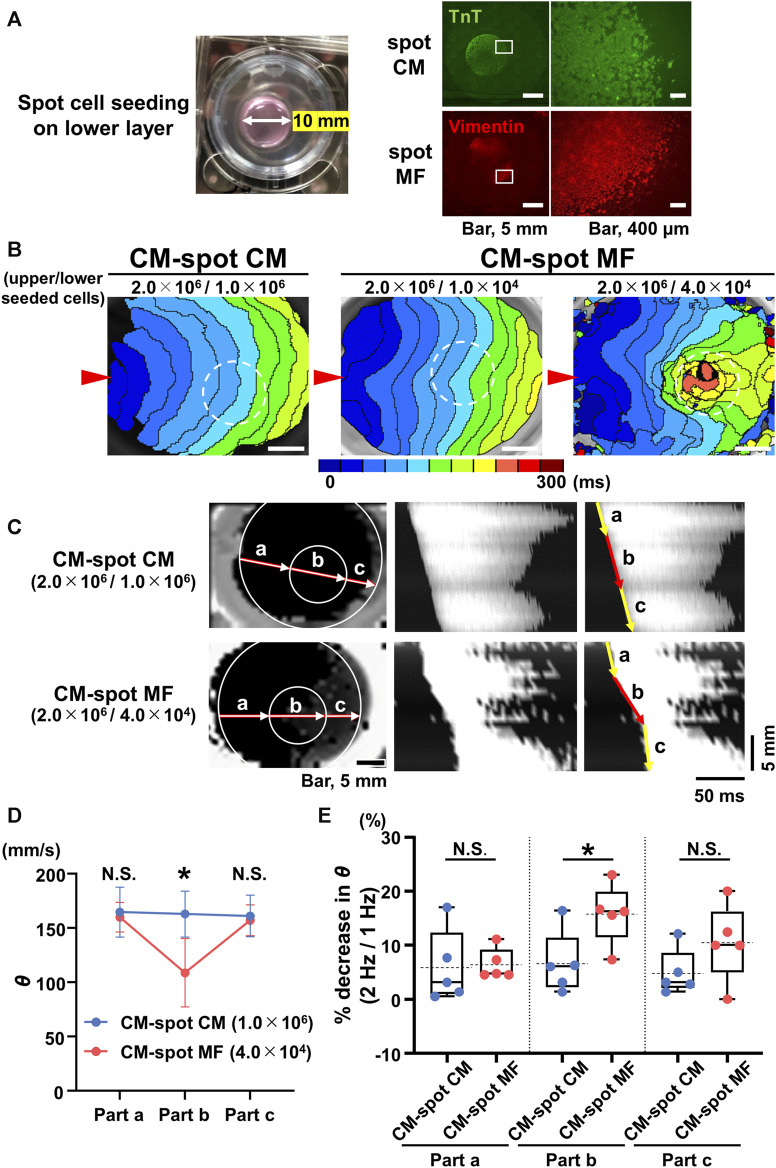
Spot seeding of the myofibroblasts (MFs) causes local propagation slowing of the cardiomyocyte (CM) monolayer. **(A)** The gross image of the spot-seeded cell cluster on the lower layer (left panel). The 2.0 × 10^6^ CMs were cultured on the upper side of the cell culture insert and either MFs (4.0 × 10^4^ cells) or CMs (1.0 × 10^6^ cells) were locally seeded on the reverse side using a 10-mm diameter silicon cylinder. The representative immunofluorescence images for the CMs and MFs locally seeded on the lower layers (right panels). The magnified images at the square portions are shown on the right side. Note that the locally cultured-cell clusters are sharply marginated. **(B)** The representative isochronal maps of the upper CM layer under local seeding of CMs (1.0 × 10^6^ cells) and MFs (1.0 × 10^4^ cells and 4.0 × 10^4^ cells) on the lower layer. Red arrowheads denote the point of electrical pacing. White dashed circles denote the region of the local cell seeding. The white scale bar = 5 mm. **(C)** The representative X-Y (left panel) and X–t (middle and right panels) fluo8-fluorescence images for the impulse propagation in the upper CM monolayer. Parts a, b, and c on the line of the X-Y image correspond to parts a, b, and c of the X-t image as illustrated by arrows. **(D)** Conduction velocity (*θ*) at parts a, b, and c in the upper CM layer under local seeding of CM (CM-spot CM) and MF (CM-spot MF) during 1-Hz pacing (CM-spot CM: n = 5/group, obtained from 3 isolations; CM-spot MF: n = 5/group, obtained from 4 isolations). Data are presented as mean ± SD. *p < 0.05. **(E)** The percent increase in conduction velocity (*θ*) as stimulus frequency rate increased from 1 Hz to 2 Hz for each part **(A–C)** (n = 5 for each group corresponding to **(D)**. Data are box-plotted as median ± quartiles (25% and 75%). Mean values on the graphs are depicted as horizontal dashed lines. *p < 0.05. N.S. = not significant.

We tested the extent to which the CM monolayer alters the spatiotemporal impulse propagation when MFs are locally cultured on the reverse side. We performed spot cell seeding on the lower layer of the cell culture inserts; the CMs (2.0 × 10^6^ cells) were cultured on the upper side, and either MFs (1.0 × 10^4^ or 4.0 × 10^4^ cells) or CMs (1.0 × 10^6^ cells) were cultured in a spot pattern on the reverse side of the cell culture insert using a 10-mm diameter silicon cylinder (Figure 3A). The isochronal map for the CM-spot MF model demonstrated a concave pattern of the wavefront of the upper CM layer in the region as compared with that for CM-spot CM model (Figure 3B), consistent with the depressed conduction velocity in the locally cultured MFs behind, and a slower *θ* than that of the surrounding area (Figures 3C, D), whereas no regional difference was observed in the *θ* value when CMs were locally cultured in the lower layer (Figures 3B–D). In addition, the regional slowing of the conduction velocity was dependent on the number of MFs; the larger the number of MFs cultured, the more remarkable was the regional slowing of the impulse propagation (Figure 3B). A similar regional depression of *θ* was observed in another preparation, where 4 × 10^4^ MFs were locally seeded in the lower layer. In the CM-spot MF model, the propagation was slowed only in the MF-cultured area on the reverse side, but not in the surrounding cell-free area (Figure 3C). The conduction velocity (*θ*) of the upper CM layer at the cell-cultured area of the reverse side (part b) was slower in the CM-spot MF preparation than in the CM-spot CM preparation (CM-spot CM: 162.9 ± 21.1 mm/s [n = 5] vs. CM-spot MF: 108.7 ± 31.6 mm/s [n = 5], *p* = 0.0127) (Figure 3D). In addition, under the local cell seeding on the reverse side, the stimulus frequency-dependent decrease in the *θ* ratio (1 Hz vs. 2 Hz) of the upper CM layer (in part b) was also larger in the CM-spot MF preparations than in the CM-spot CM ones (CM-spot CM: 6.63% ± 5.85% [n = 5] vs. CM-spot MF: 15.80% ± 5.59% [n = 5], *p* = 0.0351) (Figure 3E). There was no regional difference in the mean *θ* in part b between the CM-spot CM preparations and the CM-spot HeLa ones (CM-spot CM: 162.9 ± 21.1 mm/s [n = 5] vs. CM-spot HeLa: 158.0 ± 16.1 mm/s [n = 3], *p* = 0.744) (Supplementary Figure S2D). On the 12 CM-spot MF preparations, 7 showed spontaneous firing of the CM layer, and 4 spontaneously turned into a re-entrant propagation that surrounded the area of the MF cluster on the reverse membrane side (Supplementary Figure S3). Additionally, no re-entrant pattern was observed in the CM-spot CM preparations, in which four out of nine showed spontaneous firing. There was no significant difference observed in the probability of spontaneous firing between CM-spot CM and CM-spot MF preparations (CM-spot CM: [4 out of 9, obtained from 6 cases of isolation] vs. CM-spot MF: [7 out of 12, from 6 cases of isolation], *p* = 0.670).

### 3.3 MFs connect with CMs through pores of the culture membrane via Cx43 gap junction

**FIGURE 4:**
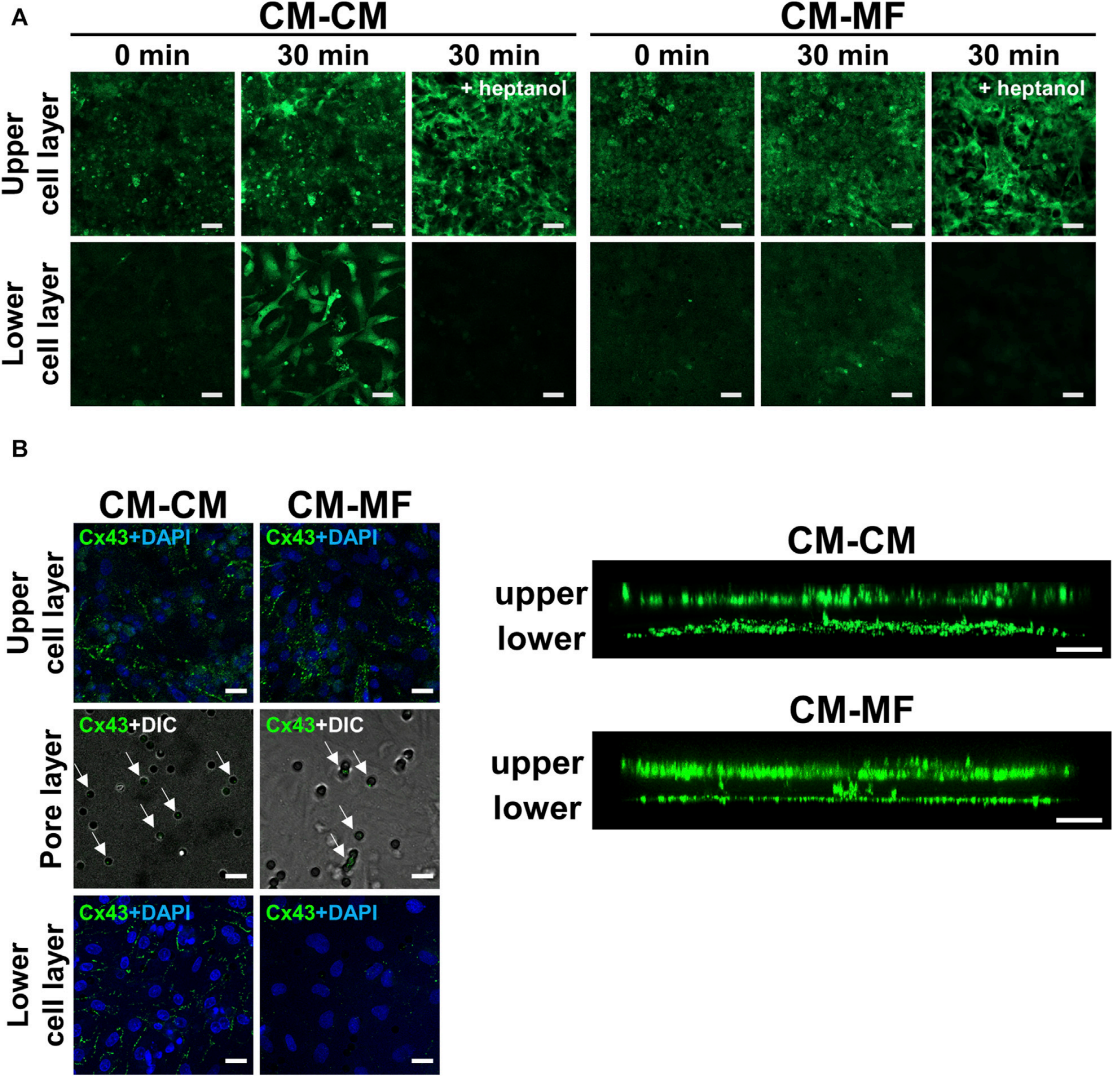
Myofibroblasts (MFs) connect with cardiomyocytes (CMs) through pores of the culture membrane by gap junction. **(A)** The representative fluorescent images of calcein on the upper and lower cell layers. The images were taken at 0 and 30 min after calcein-AM loading. The right panel was taken at 30 min after calcein-AM loading under pretreatment with 0.5 mM heptanol. The scale bar = 100 μm. **(B)** The representative confocal immunofluorescence images of Cx43 on the upper CM and lower cell (CM or MF) layers. On the pore layer, immunofluorescence images for Cx43 are overlaid with DIC images showing micropores. The corresponding side-view, stack images are shown on the right panel. The scale bar = 20 μm.

To address the mechanisms of the impaired impulse propagation of CMs in the CM-MF model, we examined whether CMs in the upper layer were functionally coupled with cells cultured on the lower side. We evaluated calcein fluorescent dye transfer in the lower cell layer 30 min after calcein-AM loading in the upper CM layer. Calcein was transferred to the lower cell layers in the CM-CM and CM-MF models (Figure 4A) but not in the CM-HeLa model (Supplementary Figure S4A). Heptanol, a gap-junctional inhibitor at 0.5 mM, completely blocked the transfer of the calcein dye to the lower cell layers in the CM-CM and CM-MF models (Figure 4A), indicating that the calcein dye transfer from the upper cell layer to the lower is mediated by gap junctions between the two layers of cells.

We also evaluated the immunocytochemical expression of Cx43 in CMs and MFs, a major gap-junction protein that connects CM with MF *in vitro* (Miragoli et al., 2006; Askar et al., 2012) and *in vivo* (Camelliti et al., 2004; Rubart et al., 2018). Both the CM and MF layers showed abundant Cx43 expression as shown in the side-view stack images (Figure 4B, right panel). Moreover, Cx43 fluorescence was detected in the pores of the cell culture membrane (Figure 4B, left panel), indicating the presence of Cx43 in between the cells in the upper and lower layers. In the CM-HeLa model, Cx43 expression was observed only in the upper CM monolayer but not in the membrane pores or lower HeLa cell layer (Supplementary Figure S4B).

### 3.4 Selective Cx43 knockdown in the MFs improves both the velocity and uniformity of impulse propagation of the CM layer

**FIGURE 5:**
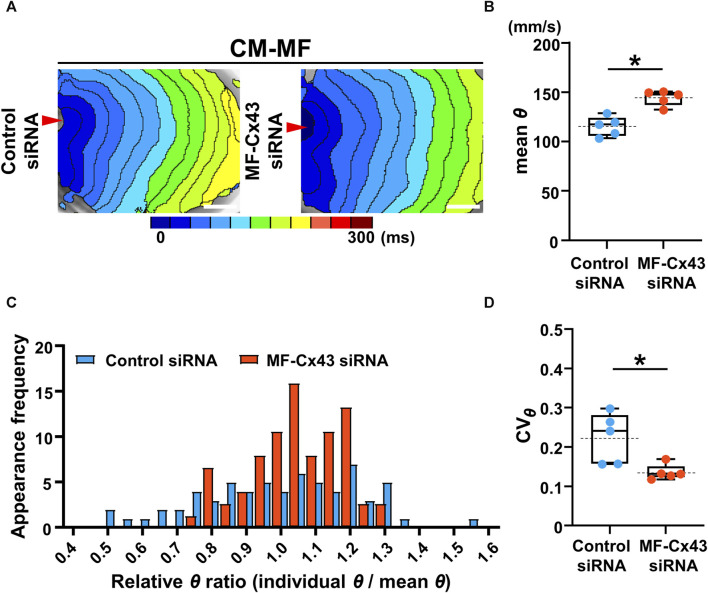
Selective Cx43 knockdown of the myofibroblasts (MFs) improves both the velocity and uniformity of impulse conduction in the cardiomyocyte (CM) monolayer. **(A)** The representative isochronal maps of CM-MF models after treatment with the control siRNA or MF-selective Cx43 siRNA. The white scale bar = 5 mm. **(B)** The mean conduction velocity (mean *θ*) on the upper CM layer of the CM-MF model during 1-Hz pacing (control siRNA: n = 5/group, obtained from 3 isolations; MF-Cx43 siRNA: n = 5/group, obtained from 4 isolations). **(C)** The histogram of relative conduction velocity ratios of CM-MF models treated with the control siRNA and MF-Cx43 siRNA. **(D)** The coefficient of variation (CV_*θ*_) for the *θ* of the upper CM layer during 1-Hz pacing (n = 5 for each group corresponding to **(B)**. Data in **(B,D)** are box-plotted as median ± quartiles (25% and 75%). Mean values are depicted on the graphs as horizontal dashed lines. *p < 0.05.

To address whether MF-selective Cx43 inhibition improves the impaired impulse propagation of the CM layer in the CM-MF model, we knocked down Cx43 in MFs using siRNA (Supplementary Figure S5). Under Cx43 knockdown of the MFs in the CM-MF model, the isochronal maps revealed that the conduction pattern of the upper CM layer (Figure 5A) restored nearly to that of the CM-CM model like as shown in the left panel in Figure 2A. The mean *θ* of the CM monolayer at 1-Hz pacing was significantly faster in the MF-Cx43 siRNA preparations than in the control siRNA preparations (control siRNA: 115.6 ± 9.9 mm/s [n = 5] vs. MF-Cx43 siRNA: 144.5 ± 7.7 mm/s [n = 5], *p* = 0.0079) (Figure 5B). In the Cx43 siRNA-treated MF preparation of the CM-MF model, the histogram of the relative *θ* ratio to the mean *θ* at 1-Hz pacing showed a smaller variation than that for the control siRNA preparation (Figure 5C), and the CV_*θ*_ for the mean *θ* value of the CM layer at 1-Hz pacing was significantly smaller than that of the control siRNA preparation (control siRNA: 0.224 ± 0.064 [n = 5] vs. MF-Cx43 siRNA: 0.136 ± 0.020 [n = 5], *p* = 0.0185) (Figure 5D).

**FIGURE 6:**
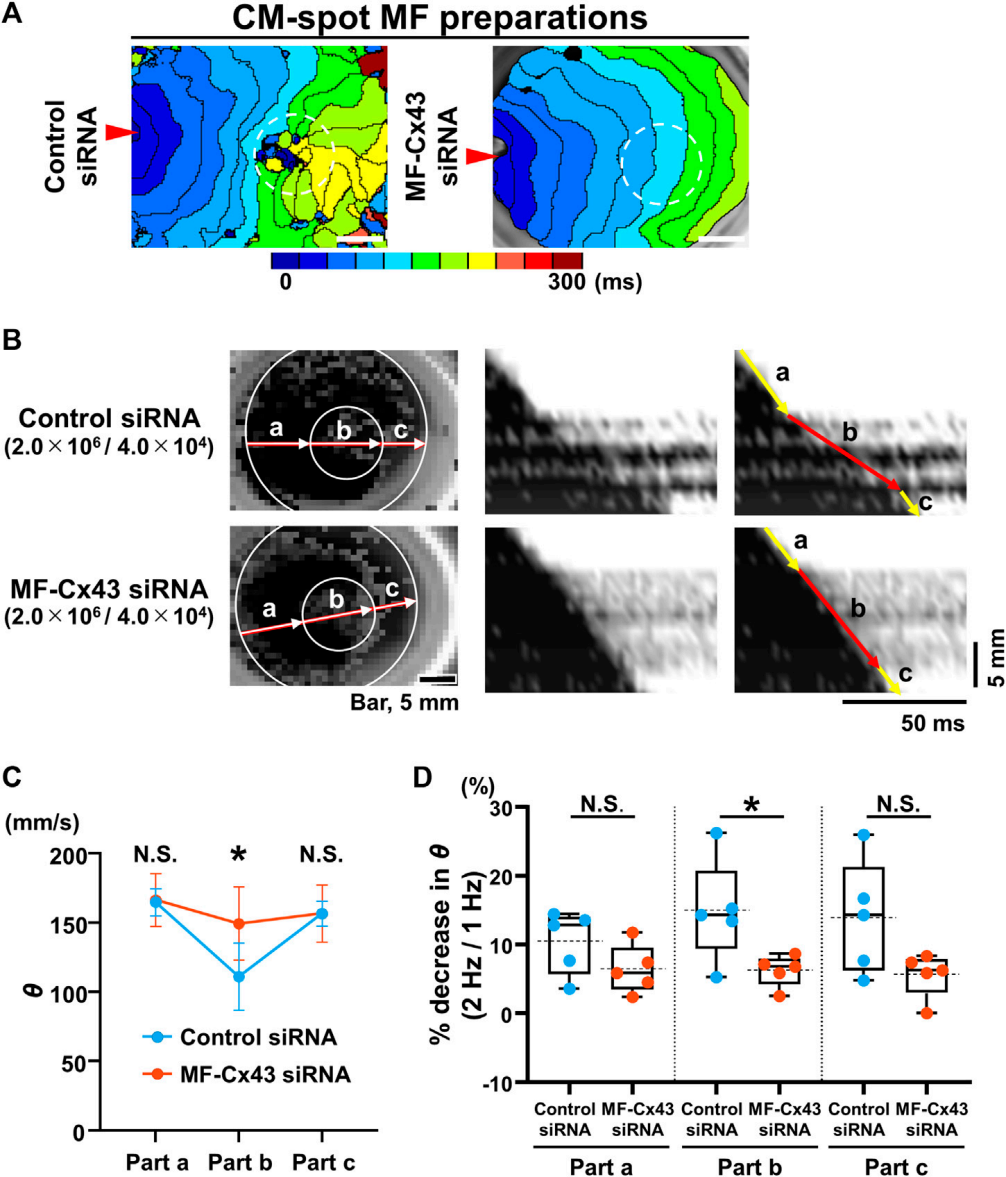
Myofibroblast (MF)-selective Cx43 knockdown improves regionally depressed impulse propagation of the cardiomyocyte (CM) layer of myofibroblast (MF) spot-seeded preparation. **(A)** The representative isochronal maps of the upper CMs layer of CM-spot MF preparations after treatment with the control siRNA and MF-Cx43 siRNA. Dashed circles indicate the site of spot placement of MFs on the lower side of the membrane. The white scale bar = 5 mm. **(B)** The representative X-Y and X-t fluo8-fluorescence images of the impulse propagation of the upper CM monolayer. **(C)** Conduction velocity (*θ*) at parts a, b, and c of the upper CM layer during 1-Hz pacing after treatment of MFs with the control siRNA and MF-Cx43 siRNA (control siRNA: n = 5/group, obtained from 2 isolations; MF-Cx43 siRNA: n = 5/group, obtained from 4 isolations). Data are presented as mean ± SD. *p < 0.05. **(D)** The percent increases in the *θ* as stimulus frequency rate increased from 1 Hz to 2 Hz for each part **(A–C)** (n = 5 for each group corresponding to **(C)**. Data are box-plotted as median ± quartiles (25% and 75%). Mean values are depicted on the graphs as horizontal dashed lines. *p < 0.05. N.S. = not significant.

We also evaluated the effect of MF-selective Cx43 knockdown on impulse propagation in the CM monolayer under the spot seeding of an MF on the reverse side. Isochronal maps of the CM-spot MF model and X-t images revealed that *θ* in the MF-cultured area was faster and more uniform in the MF-Cx43 siRNA preparation than in the control siRNA one (Figures 6A, B). During 1-Hz pacing, the *θ* of the upper CM layer at the MF-cultured area behind (part b) was significantly faster in the MF-Cx43 siRNA preparation than in the control siRNA one (control siRNA: 111.2 ± 24.1 [n = 5] mm/s vs. MF-Cx43 siRNA: 149.5 ± 26.4 mm/s [n = 5], *p* = 0.0437) (Figure 6C). The percent decrease in *θ* of the CM layer (part b) upon increasing the pacing frequency from 1 Hz to 2 Hz was significantly greater in the control siRNA preparation than in the MF-Cx43 siRNA one (control siRNA: 14.88% ± 7.47% [n = 5] vs. MF-Cx43: siRNA 6.21% ± 2.31% [n = 5], *p* = 0.0382) (Figure 6D).

## 4 Discussion

### 4.1 Summary of this study

To our knowledge, this is the first *in vitro* study on the spatiotemporal changes in the impulse propagation of the CM monolayer using a microporous membrane co-cultured with MFs on the reverse side. Using this unique experimental model, we established that the CM monolayer connects to the MFs solely via micropores. The main findings of this study are as follows: i) MFs co-cultured on the reverse side of the membrane slow and impair spatiotemporal impulse propagation in the CM monolayer, ii) local seeding of an MF cluster on the reverse membrane side generates a slow-conducting segment in the CM layer, and iii) MF-selective knockdown of Cx43 abolishes depressed impulse propagation in the CM layer. Moreover, we demonstrated for the first time that MFs directly induce conduction slowing by gap-junctional coupling alone even in the absence of mechanosensitive interactions between MFs and CMs.

### 4.2 Depressed impulse propagation in the CM-MF model

In the present study, we provide direct proof of concept for spatiotemporal alterations in CM monolayers produced by the single-sided connection with MFs. Computer simulation studies have predicted that placement of MFs over the CM monolayer reduces the conduction velocity in models of 1-dimensional CM strands (Sachse et al., 2009; Jousset et al., 2016) and 2-dimensional CM layers (Jacquemet and Henriquez, 2008; Xie et al., 2009). However, experimental studies on single sided CM-MF connection by using CM strands or CM monolayers with placement of MFs have not necessarily confirmed these predictions owing to the inability to create pure mechanical strain-free conditions. The present study demonstrated that MFs co-cultured on the opposite side of the microporous membrane slowed and impaired the spatiotemporal impulse propagation of the CM monolayer, whereas co-culture with CMs, connection-deficient HeLa cells, or no cell on the reverse side failed to impair impulse propagation. Furthermore, MF-selective Cx43 knockdown in the CM-MF model normalized the depressed impulse propagation. The definitive presence of Cx43 in the membrane pores and heptanol inhibition of calcein dye transfer from the upper to the lower layers would indicate the heterocellular Cx43 gap-junctional coupling between the two layers via the membrane pores. Taken together, our findings suggest that depressed impulse propagation in the CM-MF model is associated with a heterocellular gap-junctional coupling with MF, specifically Cx43 gap-junctional communication with no involvement of mechanical interactions.

The slowing of the conduction velocity (*θ*) in the CM-MF model can be explained by the possible electrotonic elevation in the resting membrane potentials of CMs by non-excitable MFs having more depolarized potentials than CMs (Jacquemet and Henriquez, 2008; Xie et al., 2009), resulting in inactivation of the tetrodotoxin-sensitive Na^+^ channel of CMs (Miragoli et al., 2006). Interestingly, we found a more remarkable *θ* depression at 2 Hz than at 1 Hz in CM-MF models. We have no direct electrophysiological evidence to explain this phenomenon; however, we assume that inadequate recovery of depolarizing current from inactivation may account for it. This is because a simulation study indicates slowing of the Na^+^ channel recovery from inactivation by the CM-MF coupling-induced elevation of the myocyte resting potential; retardation of Na^+^ current recovery from inactivation by CM-MF coupling arises at diastolic intervals of 300 ms and shorter (Xie et al., 2009). Thus, the CM layer can be depolarized by MFs not only leading to inactivation of Na^+^ channels but may also potentially retarding the recovery from Na^+^ channel inactivation, and thereby, frequency-dependent slowing of impulse propagation may occur. Further studies are required to verify this possibility.

Besides the resting membrane-potential effects by MFs, the relatively large size of MFs compared with that of CMs could also potentially contribute to the conduction slowing in the CM-MF model by acting as a large electrical sink; CMs of smaller capacitance than MF can be charged by coupled MFs having large capacitance (De Simone et al., 2020), resulting in a relative reduction of the depolarizing current for firing of CMs (Miragoli, et al., 2006), and thereby reducing the action potential upstroke and conduction velocity. Notably, we observed that seeding of MFs at 3 × 10^6^ cells on the reverse side, nearly identical cell number to that of the upper CMs, failed to evoke excitatory Ca^2+^ transients by electrical stimulation in all 7 preparations (obtained from 3 cases of isolation) tested, consistent with the MF-load-dependent conduction block (Xie et al., 2009). This observation suggests marked resting membrane depolarization of the CMs. Thus, in addition to the electrotonic effects, the capacitive loading of MFs on CMs (De Simone et al., 2020) would also contribute to the slowing of impulse propagation in our CM-MF model.

The irregular wavefront in the isochronal map, that is, the directionally inhomogeneous conduction in the CM-MF model observed in this study, could be explained by the spatially non-uniform CM-MF coupling. This is because the membrane micropores were distributed randomly on the cellular scale and the punctate Cx43 fluorescence was identified non-uniformly under the overall placement of the confluent MF monolayer on the membrane. Our observations are in agreement with those of the *in silico* study by Jousset et al. (2016), who demonstrated that the random attachment of MFs to the CM strand model distorts the uniformity of the wavefront, presumably owing to the spatial inhomogeneity of the resting membrane potentials of the CM monolayer (Jousset et al., 2016). In addition, inhomogeneous impulse propagation could also occur by different levels of gap-junctional coupling of the myocardium. For example, TGF-β1, a cytokine produced by MFs, was also reported to upregulate Cx43 expression and augment gap-junctional coupling conductance between CMs and MFs, which may augment the arrhythmogenic potentials of MFs (Salvarani et al., 2017).

### 4.3 Regional depression of impulse propagation of the CM layer under connection with a localized MF cluster

The local placement of an MF cluster on the reverse membrane side showed a well-circumscribed, localized depression of *θ* in the CM layer. In addition, the regionally depressed impulse propagation was dependent on the number of the spot-seeded MFs; the larger the number (approx. 4 times) of cells seeded, the more remarkably the *θ* was depressed. Moreover, the local seeding effect of the MFs also showed pacing-frequency dependence, which was more remarkable at 2 Hz than at 1 Hz. Therefore, the regional differences in *θ* between the MF-seeded region and the surrounding cell-free regions on the reverse membrane side would become more remarkable at pacing rate of 2 Hz or higher than at lower rate because the *θ* values of the surrounding region were not altered between 1 Hz and 2 Hz. Such frequency-dependent local slowing of conduction would augment regional inhomogeneity of impulse propagation by high-frequency or premature excitation and may predispose the heart to proarrhythmogenic. Thus, we assume that the local clusters of MFs in point-to-point contact with the CM tissues could become a substrate for the development of re-entrant arrhythmias. This notion is supported by a computer simulation study showing that the localized attachment of MF cluster to the CM monolayer model facilitates the inducibility of re-entrant propagation owing to the electrotonic depolarization of CMs and the resultant retardation of recovery from Na^+^ channel inactivation with prolongation of the refractory period (Xie et al., 2009). Regarding the spatial non-uniformity of Cx43 gap-junctional communication for arrhythmogenesis, regionally clustered CM-MF coupling rather than randomly distributed CM-MF coupling is more likely to contribute to the genesis of re-entrant arrhythmias, analogous to the arrhythmogenicity of the spatially non-uniform Cx43 of CMs (Gutstein et al., 2001; Nakagami et al., 2008).

### 4.4 Improvement of impaired impulse propagation by MF Cx43 knockdown

We demonstrated that the conduction slowing and non-uniformity of the wavefront in the CM-MF model were improved by the MF-selective Cx43 knockdown, indicating the essential role of Cx43-gap-junctional CM-MF coupling in impaired impulse propagation. The MF-selective Cx43 knockdown effects we observed are analogous to those of previous studies (Zlochiver et al., 2008; Askar et al., 2012); however, these studies used mixed co-culture models of CMs and MFs, distinct from our pure single-sided connection model with no mechanosensitive interactions. One may consider the possibility that the MF-selective Cx43 knockdown have also suppressed the inter-MF coupling. However, the impulse propagation of upper CM layer of our CM-MF model would not be influenced by attenuation of inter-MF coupling because the upper CM layers involve few, if any, MFs.

Our findings indicate a therapeutic potential for MF-specific Cx43 ablation against re-entrant tachyarrhythmias that may originate especially in the infarct border zone, where the single-sided CM-MF connection predominantly resides. The MF-selective Cx43 knockdown would attenuate both the resistive and capacitive couplings of MFs and CMs, thereby leading to reduce proarrhythmogenic potentials in the infarct border zone. However, we need to consider the possibility that MF-selective Cx43 knockdown involved in myocardial tissue may adversely promote the arrhythmogenicity of MFs. This is because in addition to the annihilation of the electrotonic effects in the CM-MF interface and inter-MF connections, the single-sided CM-MF connections may transform into zero-sided ones when Cx43 in the MFs is silenced, and thereby, MFs may become electrical insulators. Besides, MF-selective inhibition of Cx43 may prevent the differentiation and proliferation of MFs (Asazuma-Nakamura et al., 2009), which could retard the healing of myocardial infarction (Zhang et al., 2010). Contrary to the Cx43 inhibition, Roell et al. (2018) demonstrated that Cx43 overexpression of MFs in the border zone of mouse myocardial infarction increases conduction velocity and reduces the likelihood of arrhythmias possibly via improvement of inter-MF gap-junctional connection (Roell et al., 2018). Such interventions would be promising to improve the depressed impulse propagation and reduce the spatiotemporally inhomogeneous electrical conduction on the infarct border zone and could attenuate genesis of re-entrant arrhythmias. In conjunction with this *in vivo* study, Lagonegro et al. demonstrated a unique therapeutic intervention against arrhythmogenesis; local *in-situ* injection of multiple nanowires into the cryoinjured zone improved the depressed impulse conduction of the mouse heart (Lagonegro et al., 2022). Further studies are required to determine the feasibility of these promising strategies.

### 4.5 Pathophysiological significance of Cx43 gap-junctional connection between CMs and MFs

We demonstrated that the point-to-point, single-sided CM-MF connection impaired the impulse propagation of the CM monolayers. The single-sided CM-MF connection model was also studied using a linear connection model between monolayers of CMs and MFs with Cx43 without the insertion or mixture of these two types of cells (Zhao et al., 2020). Under such a linear interface, the authors observed the spontaneous occurrence of re-entrant arrhythmias in the CM monolayer after the application of hydrogen peroxide by prolonging the action potential duration (Zhao et al., 2020). Augmentation of the arrhythmogenic potentials of MFs by oxidative stress was also demonstrated in the rabbit ventricular myocyte coupled with virtual MF model cells (Nguyen et al., 2012), in which MF-mediated prolongation of the action potential duration led to early afterdepolarization and triggered arrhythmias by hydrogen peroxide. Together, the single-sided CM-MF coupling impairs impulse propagation of the myocardium, but an additional proarrhythmic insult, i.e., “second hit,” such as ischemia/reperfusion injury, would promote the single-sided connection-mediated arrhythmogenicity.

Among the various patterns of heterocellular interactions of MFs with CMs (Pellman et al., 2016), a regional cluster of MFs connected with CMs could arise in the border zone of the healing myocardial infarct (Sun and Weber, 2000), where granulation tissues composed of abundant MFs may form single-sided connections with adjacent myocardium (Peters et al., 1994; Matsushita et al., 1999). In practice, heterocellular Cx43-gap junctions were identified between CMs and MFs in the infarct border zone of the sheep heart (Camelliti et al., 2004); however, the distribution of Cx43 was quite sparse compared to that among CMs. In this regard, Baum et al. demonstrated that interleukin 1β produced from the infarcted heart reduced Cx43 expression, and therefore, they assume that heterocellular electrical coupling would be minor, and instead, a mechano-sensitive factor plays a major role in the MF-mediated alternation of electrical activities (Baum et al., 2012).

### 4.6 Limitations of this study

This study had some limitations. First, the CM monolayer showing a random orientation consisting of polygonal CMs with point-to-point attachment of MFs is distinct from the real heart tissue with three-dimensional, anisotropic arrangements of rod-shaped CMs. The relatively large MFs would also be distinct from the MFs that arise under pathophysiological conditions *in situ*. The possible cause of the large MFs can be explained by the long incubation time of 11 days in our case. Ideally, as have hitherto been reported, cultured cardiomyocytes studies derived from induced pluripotent stem cells (iPSCs) (Kadota et al., 2013; Li et al., 2020; Slotvitsky et al., 2020), especially those from humans in matured forms, would be feasible for studying the interactions between CMs and MFs in future. Second, we did not measure membrane potentials; therefore, no supporting evidence is available regarding electrophysiological alterations in CMs coupled with MFs. Third, we have no direct data on the paracrine effects of MFs and CMs, which may alter impulse propagation (Pellman et al., 2016). In this regard, our CM-MF co-culture model would negate this possibility because MFs reportedly show paracrine effects only by the culture media obtained in the CM-free conditions (Pedrotty et al., 2009; Yacoub et al., 2015). In addition, the involvement of MF paracrine mechanisms is unlikely because of i) the abrupt changes in *θ* on the MF-attached border, in contrast to gradual changes that may occur by the diffusion of paracrine substances to the surrounding area, and ii) the exclusive annihilation of the observed changes by MF-selective Cx43 knockdown. Whether the MF-selective Cx43 knockdown influences the production of paracrine factors in MFs remains unknown. Finally, we have used a limited number of preparations in this study for understanding the detailed mechanistic basis. Despite these limitations, our *in vitro* study has an impact on the arrhythmogenic potential of regional point-to-point heterocellular CM-MF coupling in the genesis of re-entrant arrhythmias. Further experimental studies are required to evaluate the impact of single-sided adjoining connections between CMs and MFs in the heart.

## 5 Conclusion

MFs can modulate patterns of impulse propagation even in the absence of their structurally intervening insulations, mechanosensitive actions, and probably paracrine actions in the myocardium. Furthermore, a single-sided CM-MF connection via Cx43-based gap junctions may play a substantial role in this modulation.

## Data Availability

The raw data supporting the conclusion of this article will be made available by the authors, without undue reservation.

## Abbreviations

CM, cardiomyocyte; MF, myofibroblast; *θ*, conduction velocity; CV_*θ*_, coefficient of directional variation for *θ*.
