# Supplementary material for: Myofibroblasts impair myocardial impulse propagation by heterocellular connexin43 gap-junctional coupling through micropores
Source: Front Physiol. 2024 Feb 23;15:1352911. doi: 10.3389/fphys.2024.1352911 (PMC10920281; doi:10.3389/fphys.2024.1352911)
Supplement: Supplementary file 1 [file DataSheet1.DOCX]

**Supplementary Information**

**Myofibroblasts impair myocardial impulse propagation by heterocellular connexin 43 gap-junctional coupling through micropores**

Yumika Tsuji, Takehiro Ogata, Kentaro Mochizuki, Shoko Tamura,

Yuma Morishita, Tetsuro Takamatsu, Satoaki Matoba, Hideo Tanaka


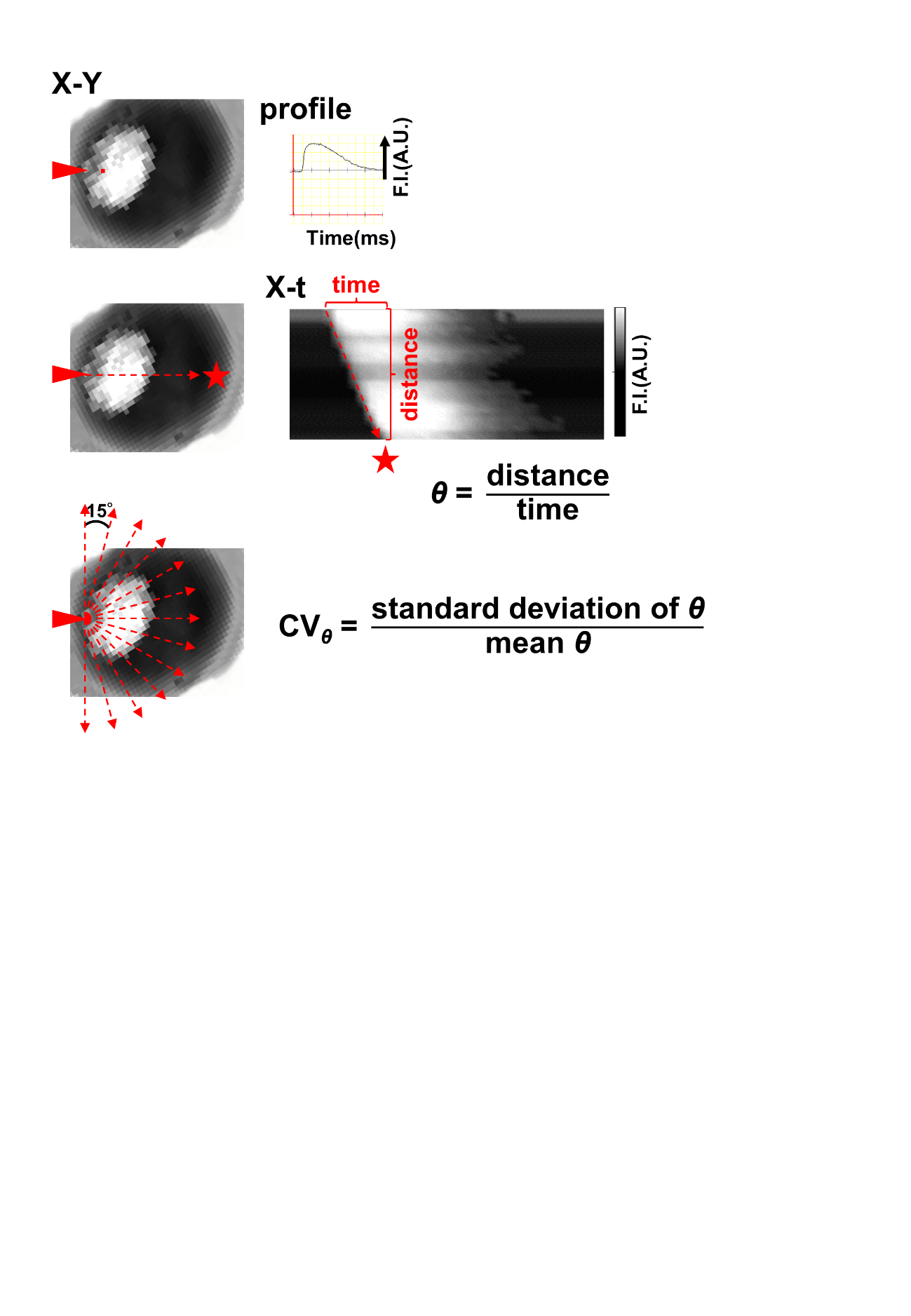


**Supplementary Figure S1.** Methods used to analyse impulse propagation of cardiomyocyte (CM) monolayers. (Top) The representative snapshot (X-Y) image of fluo8 fluorescence (left panel) during impulse propagation of the upper CM monolayer. The red arrowhead denotes the stimulation point. On the right panel, Ca^2+^ transient shown as a fluo8-fluorescence-based intensity profile at the point shown on the X-Y image. The red vertical line, the time point of stimulation. (Middle) The representative X–t image scanned in a dashed line along the direction of the wavefront of the X-Y movie. The propagation velocity (*θ*) was calculated from the slope of the X-t image. (Bottom) Illustration of the measurement methods for mean propagation velocities at 15-degree intervals. Red arrowheads denote the points of stimulation.


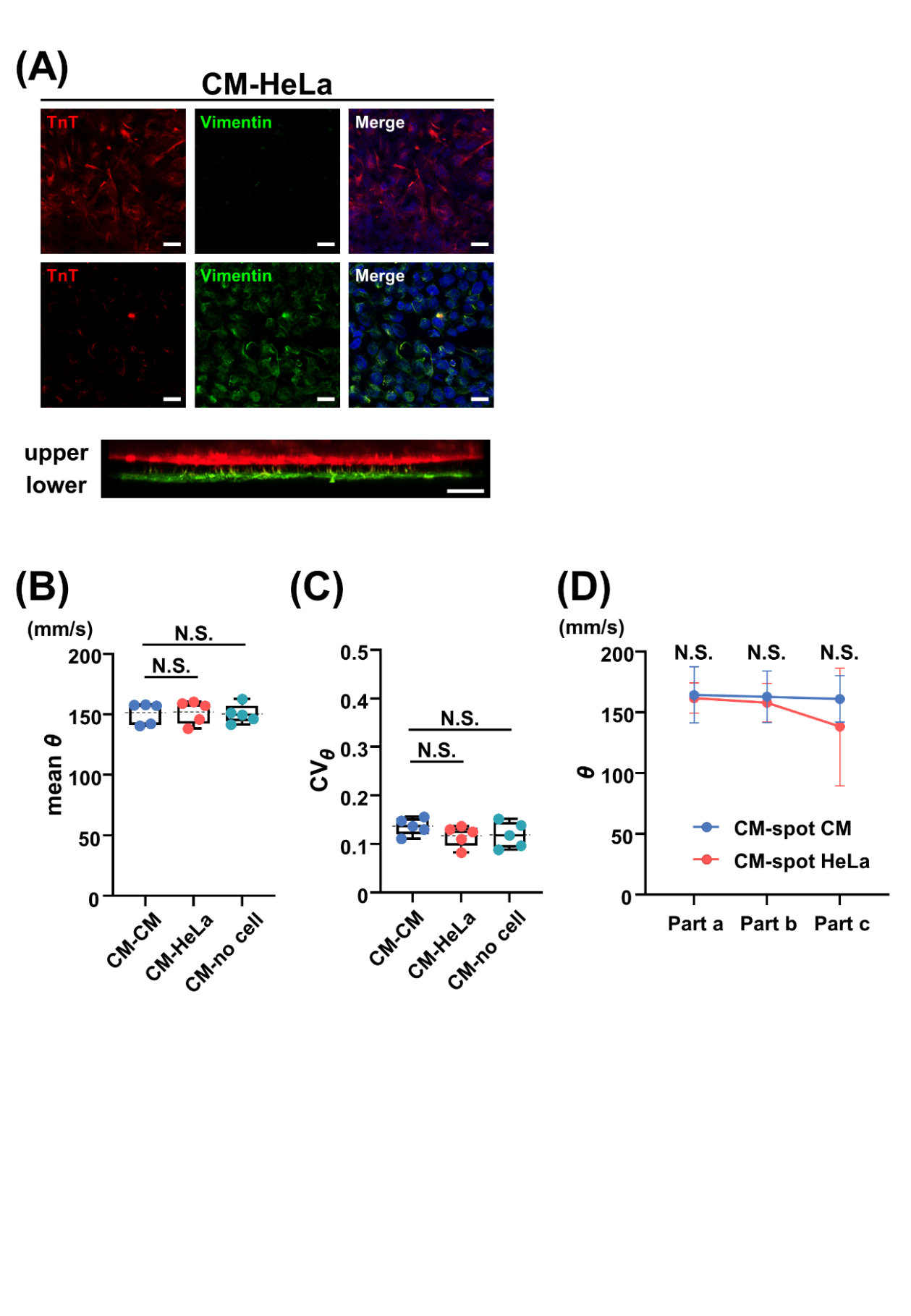


**Supplementary Figure S2.** (A) The representative immunofluorescence images of the upper cardiomyocyte (CM) and lower HeLa cell layers cultured on the microporous membrane. Lower panels show the corresponding side-view, stack image. The scale bar = 20 μm. The mean propagation velocity (mean *θ*) (B) and the coefficient of variation (CV*_θ_*) of the *θ* (C) on the upper cell layer during 1-Hz pacing in the CM-CM, CM-HeLa, and CM-no cell models (CM-CM: n=5/group, obtained from 4 isolations; CM-HeLa: n=5/group, from 4 isolations; CM-no cell: n=5/group, from 3 isolations). The data for CM-CM in (B) an (C) are identical to those shown in Figures 2B and 2E, respectively. (D) The propagation velocity (*θ*) at parts a, b, and c (in line with a, b, and c in Figures 3C and 6B) on the upper cell layer during 1-Hz pacing in the CM-spot CM (n=5/group, from 3 isolations) and CM-spot HeLa models (n=3/group, from a single isolation). Data are presented as mean ± SD. **P < 0.01. N.S. = not significant.


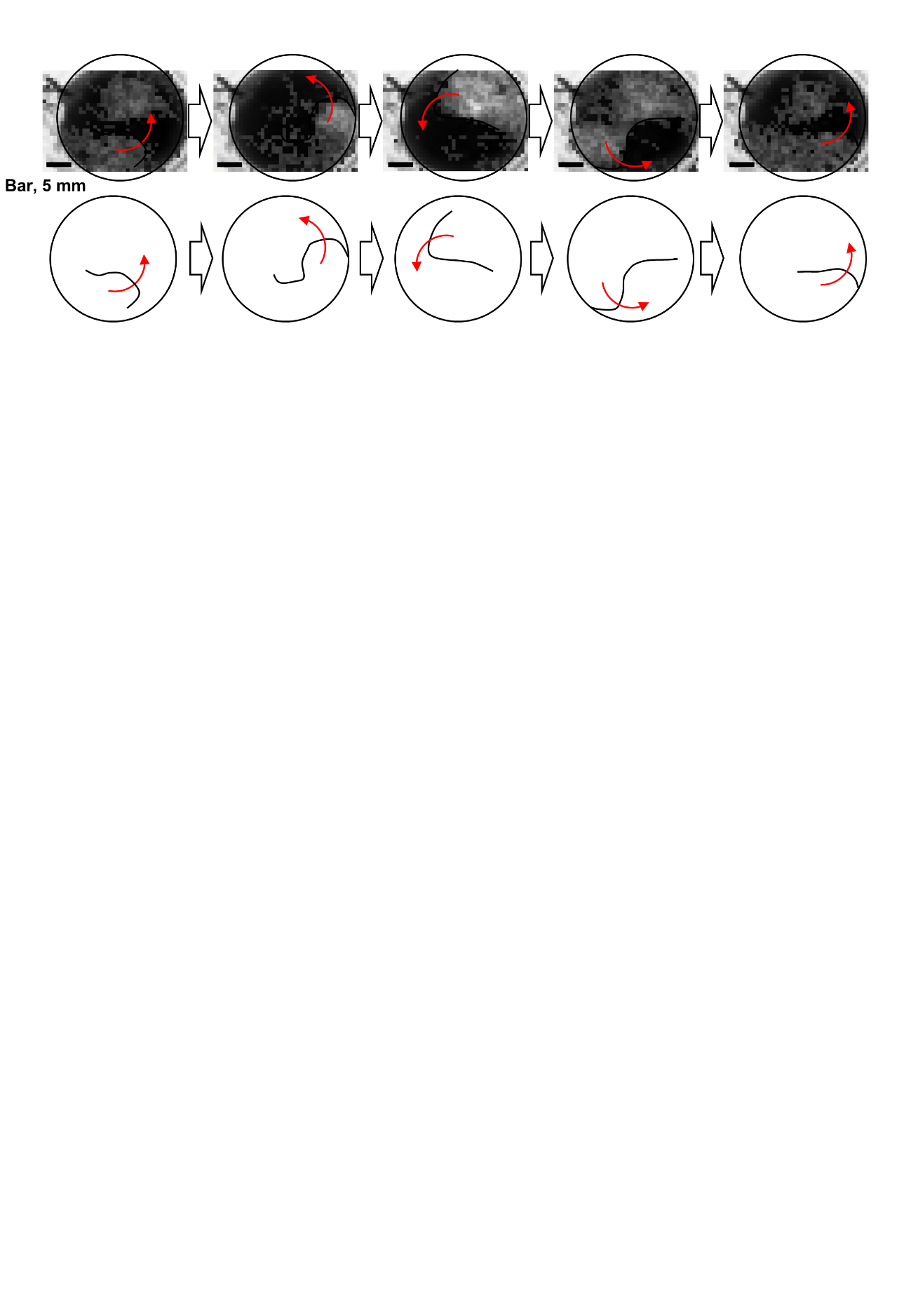


**Supplementary Figure S3.** Sequential X-Y fluo8-fluorescence images (shown every 105 ms) of a representative re-entrant propagation pattern generated in a spot seeding of a cluster of myofibroblasts (MFs) on the lower membrane layer (upper panel). The corresponding schematic drawings are shown below the fluorescence images (lower panel).


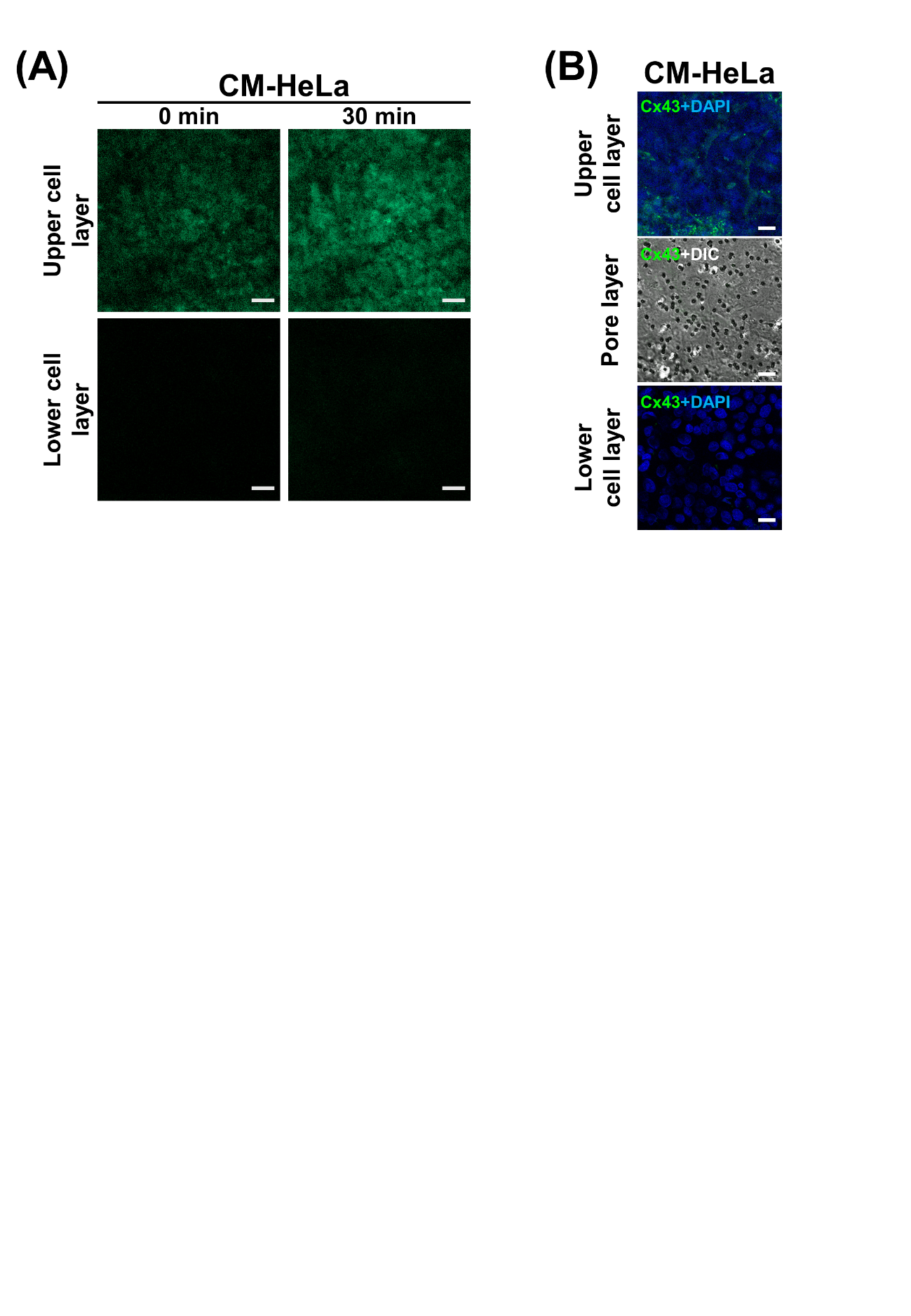


**Supplementary Figure S4. (A)** Representative fluorescence images of calcein on the upper and lower cell layers. The images were taken at 0 and 30 min after calcein-AM loading. The scale bar = 100 μm. **(B)** Representative immunocytochemical images of Cx43 on the upper cardiomyocyte (CM), pore, and lower HeLa cell layers. The scale bar = 20 μm.


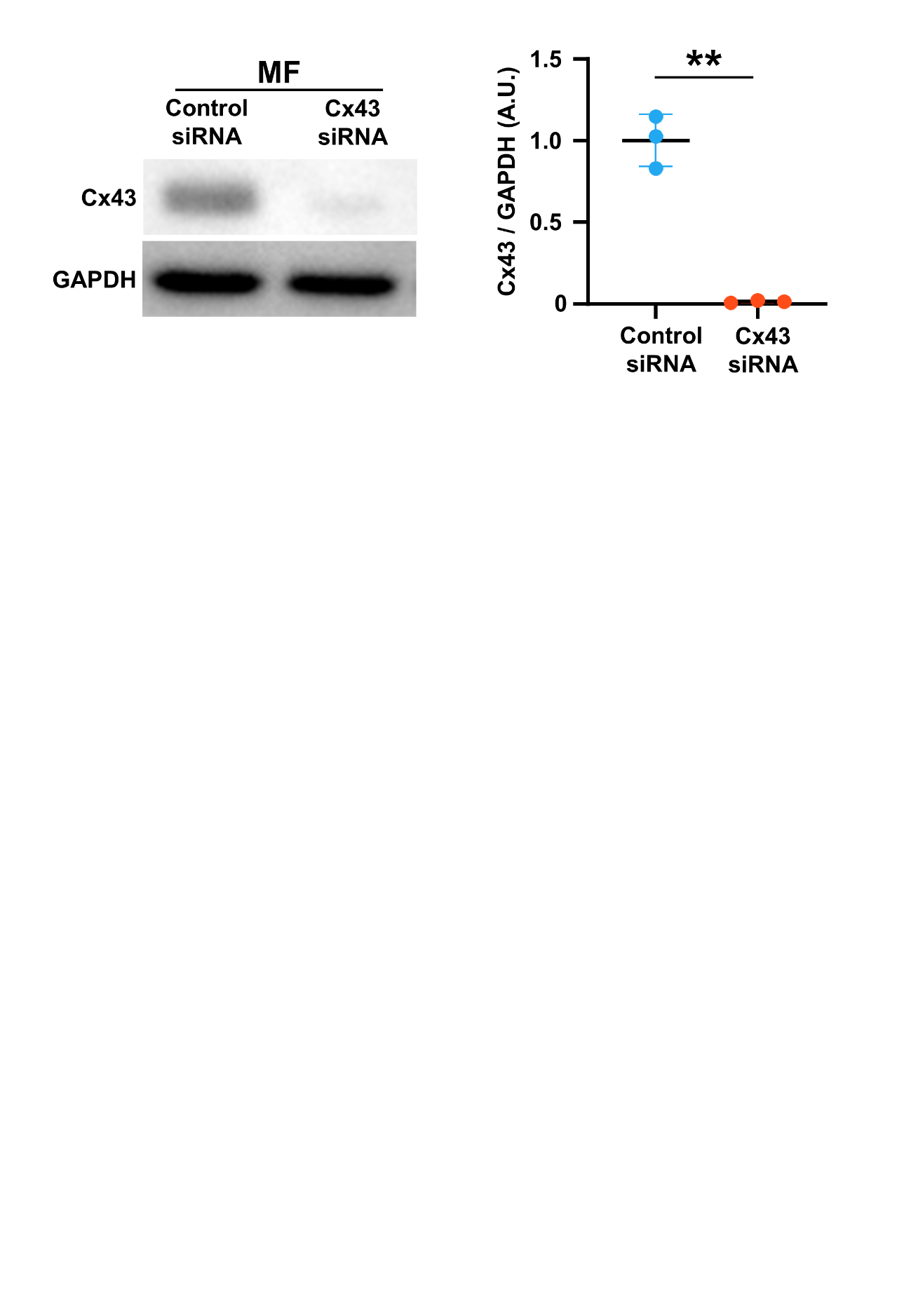


**Supplementary Figure S5.** Representative western blot image and quantitative data of the protein expression of Cx43 in myofibroblasts (MFs) after transfection of control siRNA and myofibroblast-specific Cx43 siRNA (n = 3 for each group). Data are presented as mean ± SD. ***P* < 0.01.
